# The Mysterious Risk of Arterial Thrombosis With COVID-19: A Case Series and Systematic Review of Acute Limb Ischaemia

**DOI:** 10.7759/cureus.25080

**Published:** 2022-05-17

**Authors:** Isabelle Sanders, Philip Stather, Pavithira Sivagangan, Wissam Al-Jundi

**Affiliations:** 1 Norwich Medical School, University of East Anglia, Norwich, GBR; 2 Vascular Surgery, Norfolk and Norwich University Hospitals NHS Foundation Trust, Norwich, GBR

**Keywords:** covid-19, embolectomy, thrombectomy, acute limb ischaemia, arterial thrombosis

## Abstract

Introduction: Coronavirus disease 2019 (COVID-19) generates a cytokine storm that predisposes patients to systemic complications including arterial thrombosis (AT) and acute limb ischaemia (ALI). This study reviews our understanding of the incidence and outcomes of patients with COVID-19 who develop AT.

Methods: The case notes of all emergency patients with COVID-19 referred to the vascular services between March 2020 and March 2021 were reviewed. The study was undertaken to measure 30-day outcomes. Additionally, a literature search was undertaken according to the Preferred Reporting Items for Systematic Reviews and Meta-Analyses (PRISMA) guidelines using the following search terms: acute limb ischaemia OR arterial thrombosis OR thrombectomy OR embolectomy AND COVID-19.

Results: During March 2020 and March 2021, 60,000 people tested positive for COVID-19 in Norfolk. A total of 33 patients were referred to the vascular services, of which 15 had AT (estimated incidence of 0.03%). Of AT patients, 93% had ALI. Fourteen locations of AT were identified. Of ATs, 36% were infrainguinal. The 30-day mortality was 60%. Three patients underwent surgery: two embolectomies (one requiring subsequent below-knee amputation (BKA) and the other died) and one primary BKA requiring subsequent above-knee amputation. The 30-day amputation-free survival (AFS) rate was 29%. The literature search identified 361 studies prior to a thorough full-text review. Nine case series were included with more than 10 participants each. The incidence of AT was reported as high as 15%. In-hospital mortality was 40%, with a significant proportion undergoing amputation or palliative care. Approximately a third of patients undergoing revascularisation subsequently re-occluded. AFS remained as low as 25%.

Conclusion: The incidence of AT within the vascular surgery territory in COVID-19 patients is low; however, it is associated with poor 30-day AFS. A computed tomography angiography protocol including the entire major vessels may be indicated in COVID-19 patients.

## Introduction

Severe acute respiratory syndrome coronavirus 2 (SARS-CoV-2) is a viral illness, which has the tendency to predispose patients to vascular emergencies including arterial thrombosis (AT) and acute limb ischaemia (ALI). ALI affects around 14/10,000 individuals per year and composes 16% of the vascular workload, with AT being the most challenging subtype to treat [1]. The causes of ALI can be classified into embolisation, thrombosis in situ, and, less commonly, trauma [2].

Respiratory symptoms and complications dominate the presentation of coronavirus disease 2019 (COVID-19); however, recent studies have shown the pathogenesis of the disease is more of a hypersensitivity pneumonitis as opposed to viral pneumonia. It is now more widely understood as a systemic disease. The exact mechanism of ALI in patients with COVID-19 is not yet well understood, but several theories have been proposed. It is thought that a cytokine storm activates coagulation factors and inhibits fibrinolysis, causing a hypercoagulable state. In addition to this, COVID-19 invades human cells through angiotensin-converting enzyme 2 (ACE2) receptors, leading to endothelial damage from inflammation and, subsequently, coagulation [3]. Hospital immobilisation, ICU stay, vasopressors​, and hypoxia are all risk factors contributing to Virchow’s triad (stasis, endothelial damage, and a hypercoagulable state), increasing the risk of thrombosis.

Thrombotic events including venous thromboembolism (VTE) and AT following alternative viral infections are uncommon, yet not unheard of. A retrospective study of 46 critically ill patients with the severe acute respiratory syndrome (SARS), a virus which belongs to the same *Coronaviridae* family as COVID-19, showed 11 episodes of deep vein thrombosis (DVT) and seven episodes of proven or suspected pulmonary embolism (PE) in the cohort [4]. Similarly, influenza is another virus that has proceeded to reports of thrombosis. A retrospective chart review of 119 patients admitted to the hospital with influenza found seven patients experiencing vascular events [5]. More recently, there has been growing evidence in literature reflecting the relationship between COVID-19 and thromboembolic disease.

Recognising the presence of AT in patients with COVID-19 is important as it prevents mortality and amputation rates through early intervention. Current guidelines recommend anticoagulation with heparin as the management of choice in patients with AT as it has shown to decrease mortality [6]. However, as there has been less attention to the vascular complications of COVID-19, establishing perfusion in time may prove difficult, so patients may subsequently receive limb salvage, amputation, or palliative care.

There is currently limited published data regarding the vascular complications of COVID-19. This study reviews our understanding of the incidence, management, and outcomes of patients with COVID-19 who develop thrombotic complications, to reduce direct mortality.

## Materials and methods

Case series

A retrospective case series was performed at the Norfolk and Norwich University Hospital Foundation Trust, a tertiary referral centre. The case notes of all emergency patients diagnosed with COVID-19 who were referred to the vascular services between March 2020 and March 2021 were extracted from hospital medical records and reviewed. This includes hospitalised patients. All cases of COVID-19 were tested with a polymerase chain reaction (PCR) swab and diagnosed as per hospital policies. The study was undertaken to measure 30-day outcomes.

The recorded variables included demographic characteristics, past medical history, date of admission, the reason for admission, date of a positive COVID-19 test result, presence of symptoms, arterial imaging findings, management, and mortality. Previous computed tomography angiography (CTA) imaging was used for evaluation. The indication for admission was categorised into COVID-19 and ALI (including upper and lower limb disease); patients who were admitted with stroke or coronary thrombosis were excluded. Management was categorised into anticoagulation, embolectomy, amputation, and palliation. Postoperative complications, as well as the cause of death, were recorded in relevant patients.

Systematic review

A systematic review of case series investigating the incidence and outcomes of COVID-19 and AT was undertaken using PubMed. The following search terms were used: acute limb ischaemia OR arterial thrombosis OR thrombectomy OR embolectomy AND COVID-19. Abstracts of all articles identified were reviewed, as well as articles were reviewed for additional citations. Only studies reporting solely on COVID-19-positive patients were included, with evidence of AT or ALI. Case reports were excluded, as were case series of less than 10 participants. Further studies were excluded following a full-text review.

All duplicate studies, articles reviewing coronary or intracranial thrombosis, and studies involving less than 10 participants were excluded. During the screening, data for publication date, demographic characteristics, interventions, outcomes (calculated amputation-free survival (AFS) with mortality rate), and loss to follow up were recorded by two independent reviewers (IS and PS). This was undertaken independently with discrepancies discussed with WAJ. Data extracted included the number of participants, the number undergoing revascularisation, the reintervention rate, and the number of amputations. In addition, the mortality and AFS were recorded.

Quality assessment

There was no risk of bias assessment possible; however, the studies were assessed for quality using the Joanna Briggs Institute (JBI) Critical Appraisal Checklist for case series [7]. This consists of a 10-item scale to determine the possibility of bias in the design, conduct, and analysis of each study. We included questions evaluating the inclusion criteria, methodology, completeness of inclusion, reporting of patient demographics, clinical information, and results, as well as the appropriateness of the statistical analysis.

Statistical analysis

The systematic review and data from the case series were analysed using Microsoft Excel (Microsoft, Redmond, WA) and presented as percentages. There were no comparative data; therefore, no statistical analyses were undertaken.

## Results

Case series

**Figure 1:**
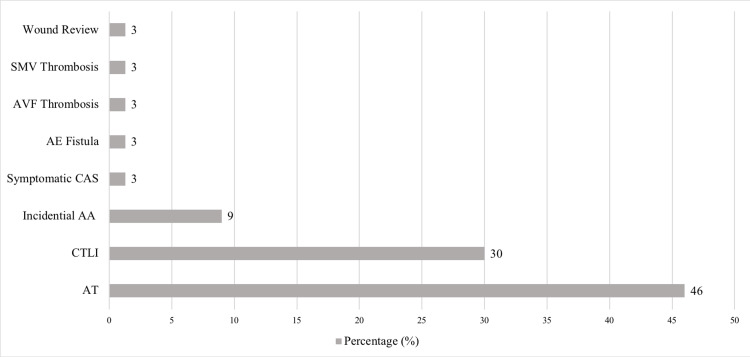
Patients with positive COVID-19 and reason for referral to vascular services. SMV, superior mesenteric vein; AVF, arteriovenous fistula thrombosis; AE, aortoesophageal fistula; CAS, carotid artery stenosis; AA, aortic aneurysm; CTLI, chronic threatening limb ischaemia; AT, arterial thrombosis.

**Figure 2:**
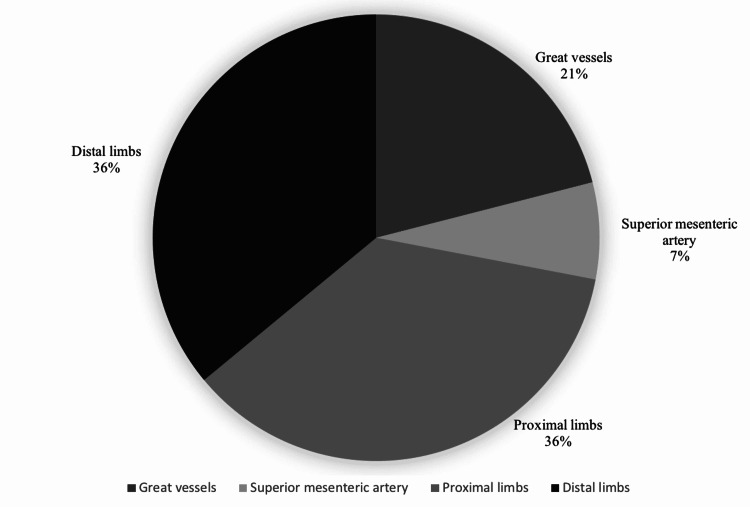
CT angiography findings of thrombus location.

A total of 33 COVID-19-positive patients were referred to vascular services between March 2020 and March 2021. Fifteen patients had acute AT (46%), which was significantly higher than any other vascular complication (Figure 1). Other presentations that did not involve AT comprised chronic limb-threatening ischaemia (10, 30%), incidental findings of abdominal aortic aneurysm (3, 9%), symptomatic carotid artery stenosis (1, 3%), fistula (1, 3%), renal access thrombosis (1, 3%), superior mesenteric vein thrombosis (1, 3%), and a postoperative wound (1, 3%). Of the patients with AT, 93% had ALI. Fourteen locations of AT were identified in nine patients (Figure 2). A total of 36% were infrainguinal, with the majority affecting the chest, abdominal, and pelvic vessels.

Within this time, there were 60,000 cases of COVID-19 in Norfolk (catchment area of 1.1 m) [8]. Out of the 33 cases referred to the vascular services, 23 (70%) were referred directly to the hub hospital and 10 (30%) were referred from other remote hospitals. As 15 patients were referred with AT, the estimated incidence of symptomatic AT was 0.03%. Therefore, three of 10,000 patients with COVID-19 have complications of AT.

Baseline characteristics are presented in Table 1. Of the patients with AT, eight were women (53%) and seven were men (47%). Their mean age at diagnosis was 72 years (range, 49-92) and 11 patients (73%) were hospitalised with COVID-19. There was a high incidence of comorbidities within the sample: atrial fibrillation (6, 40%), diabetes (4, 27%), chronic obstructive pulmonary disease (COPD)/asthma (4, 27%), ischaemic heart disease (2, 13%), hypertension (2, 13%), and chronic kidney disease (2, 13%). Only one patient (7%) had no comorbidities.

**Table 1:** Demographics and comorbidities of patients admitted with arterial thrombosis and positive COVID-19.

Demographics	Mean (range) or No. (%)
Age	72 (49-92)
Females	8 (53%)
Diabetes mellitus (DM)	4 (27%)
Atrial fibrillation/aortic valve replacement	6 (40%)
Chronic obstructive pulmonary disease (COPD)/asthma	4 (27%)
Ischaemic heart disease (IHD)	2 (13%)
Hypertension (HT)	2 (13%)
Chronic kidney disease (CKD)	2 (13%)

The 30-day mortality rate for patients with COVID-19 and AT was 60%. The direct primary cause of death was COVID-19 in 67% of these patients. Eight patients were managed conservatively with anticoagulation (53%) and five patients were palliated (33%). Of the remaining three patients, one underwent primary below-knee amputation (BKA), and two patients (13%) underwent embolectomies (one required subsequent BKA and the other died). The 30-day AFS rate was 29% (Table 2).

**Table 2:** Summary of limb amputation or death in COVID-19 patients with the presence of acute limb ischaemia.

30-day outcome	No. (%) (n = 14)
Mortality	8 (57%)
Amputation	2 (14%)
Amputation-free survival	4 (29%)

Systematic review

**Figure 3:**
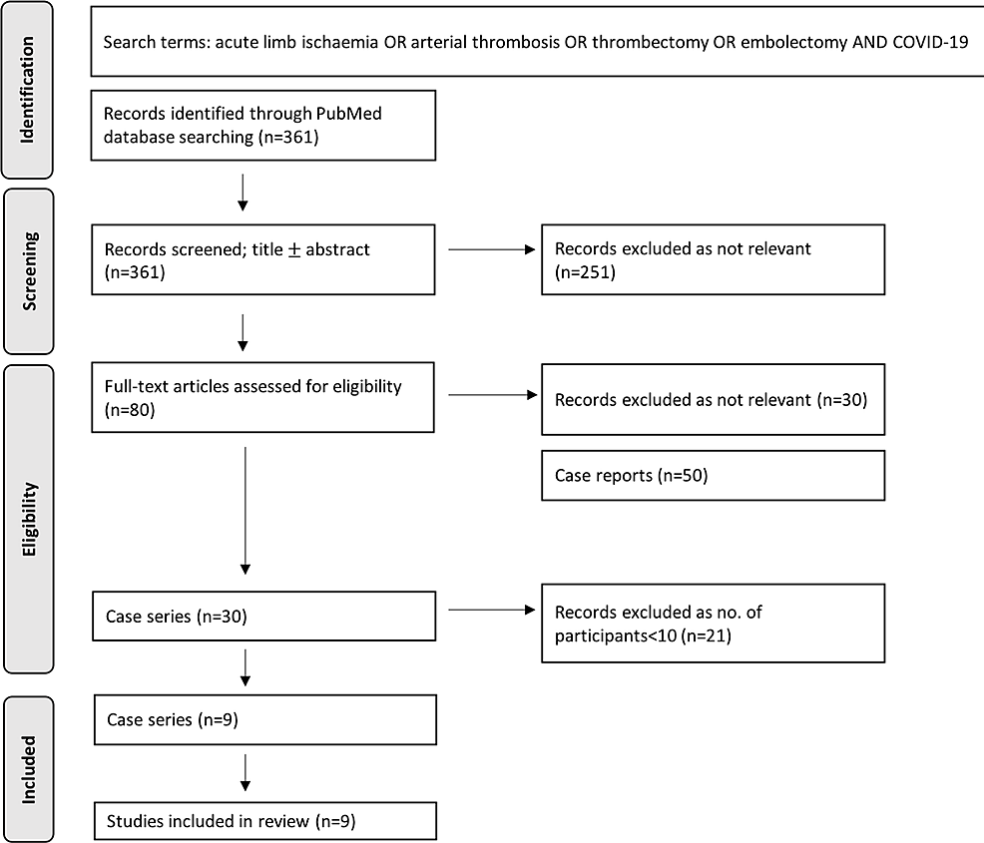
Preferred Reporting Items for Systematic Reviews and Meta-Analyses (PRISMA) flow diagram.

The literature search produced 361 results. A total of 251 studies were removed following abstract review, with a further 30 studies excluded after full-text review. A total of 21 articles with a sample size of less than 10 were excluded, resulting in nine case series. The Preferred Reporting Items for Systematic Reviews and Meta-Analyses (PRISMA) flow diagram presented in Figure 3 outlines the search results. Table 3 shows the characteristics of individual studies.

**Table 3:** Summary and characteristics of the nine studies included in this systematic review.

Name of paper	No. of participants (+ve COVID-19 + arterial events)	Arterial thrombosis (%) (arterial events (AE)/acute limb ischaemia (ALI)/arterial thrombotic event (ATE))	Amputation	Amputation-free survival rate	Mortality rate
Bellosta et al. [9]	20		1 patient	55% (11/20)	40% (8)
Goldman et al. [10]	16	100% (16/16; 16 patients with ALI and COVID-19 +ve) (94% of thrombi were proximal)	4 patients	37.5% (6/16)	38% (6)
Ilonzo et al. [11]	16	76.2% (16/21; 21 patients with acute thrombotic events and COVID-19 +ve, 15 ATE)	4 patients	56.3% (9/16)	33.3% (5)
Indes et al. [12]	13	32.5% (13/40; 40 patients with arterial thrombus, 13 COVID-19 +ve and peripheral ATE)	4 patients	15% (2/13)	40% (7)
Mascia et al. [13]	12	38.7% (12/31; 31 patients with acute limb ischaemia, 12 COVID-19 +ve patients)	2 patients	83.3% (10/12)	12.5%
Sánchez et al. [14]	30		9 patients	60% (18/30)	23.3% (7)
Tan et al. [15]	13	12.0% arterial thrombotic events in ICU (13/108; 108 all patients inc. no thrombotic events, 13 ATE), 65.0% arterial thrombotic events in ICU (13/20; 20 patients with a thrombotic event and COVID-19 +ve, 13 ATE)			Odds ratio: 4.450
Al Raizah et al. [16]	14	8.4% arterial events in ICU (inc. no thrombotic events), 2.2% (14/636; 636 patients COVID-19 +ve, 14 AE), 53.8% (14/26; 26 patients with thrombotic events and COVID-19 +ve, 14 AE)			
Gonzalez-Fajardo et al. [17]	13	12.3% (13/106; 106 patients with vascular thrombosis and COVID-19 +ve, 13 peripheral arterial thromboses)	1 patient	23.1% (3/13)	36% (9/25)
Total	147 participants		25/47 17%	47.1% on average	31.9% on average

In the selected case series, each had a sample size of 12 to 30 patients, totalling 147. The incidence of AT differed between studies ranging from 12.3% to 76.2% depending on the study population. The incidence of AT in the intensive care unit (ICU) was reported as high as 12%.

In-hospital mortality was reported in up to 40% of cases with COVID-19 and ALI, with a mean mortality rate of 31.9%. The palliation rate among the sample reached 13 patients (8.84%). A total of 80 revascularisations took place with 13 reinterventions, predominately occurring due to re-occlusion of the artery. This led to a calculated reintervention rate of 16.3% across seven studies (after excluding two with no surgical interventions). Furthermore, a significant proportion of patients underwent amputation (17%), with an average AFS rate of 47.1%. This includes both primary and secondary amputations.

Quality assessment

**Figure 4:**
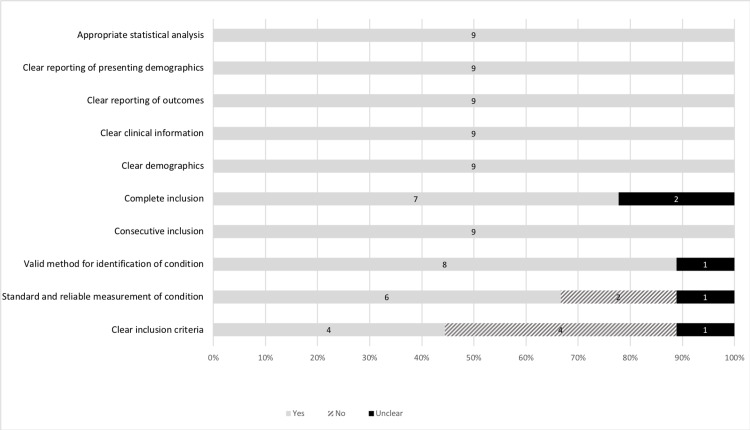
Percentage of case series that received a "yes", "no", or "unclear" for each section in the quality assessment. The number of case series is labelled within the bar.

Among the case series, four studies received a perfect score (10/10) and one study had the lowest score (6/10), with an overall mean score of 8.78 (Table 4). Having clear inclusion criteria was the lowest scoring criteria in the quality assessment (4/9, 45%). Eight criteria scored 9/9 (100%). This included consecutive inclusion, clear demographics, clear clinical information, clear reporting of outcomes, and presenting demographics, as well as appropriate statistical analysis (Figure 4).

**Table 4:** Quality assessment of individual case series. U/C, unclear.

	Were there clear criteria for inclusion in the case series?	Was the condition measured in a standard, reliable way for all participants included in the case series?	Were valid methods used for identification of the condition for all participants included in the case series?	Did the case series have consecutive inclusion of participants?	Did the case series have the complete inclusion of participants?	Was there clear reporting of the demographics of the participants in the study?	Was there clear reporting of clinical information of the participants?	Were the outcomes or follow up results of cases clearly reported?	Was there clear reporting of the presenting site(s)/clinic(s) demographic information?	Was statistical analysis appropriate?	Total
Bellosta et al. (2020) [9]	No	U/C	U/C	Yes	U/C	Yes	Yes	Yes	Yes	Yes	6
Goldman et al. (2020) [10]	No	No	Yes	Yes	Yes	Yes	Yes	Yes	Yes	Yes	8
Ilonzo et al. (2020) [11]	Yes	Yes	Yes	Yes	Yes	Yes	Yes	Yes	Yes	Yes	10
Indes et al. (2021) [12]	U/C	Yes	Yes	Yes	Yes	Yes	Yes	Yes	Yes	Yes	9
Mascia et al. 2020 [13].	Yes	Yes	Yes	Yes	Yes	Yes	Yes	Yes	Yes	Yes	10
Sánchez et al. (2021) [14]	N	N	Yes	Yes	U/C	Yes	Yes	Yes	Yes	Yes	7
Tan et al. (2021) [15]	N	Yes	Yes	Yes	Yes	Yes	Yes	Yes	Yes	Yes	9
Al Raizah et al. (2021) [16]	Yes	Yes	Yes	Yes	Yes	Yes	Yes	Yes	Yes	Yes	10
Gonzalez-Fajardo et al. (2020) [17]	Yes	Yes	Yes	Yes	Yes	Yes	Yes	Yes	Yes	Yes	10

## Discussion

This case series and systematic review demonstrate the substandard 30-day outcomes for patients who develop AT as a complication of COVID-19. The case series illustrates a high 30-day mortality rate (57%), accompanied by a low AFS rate (29%). These results are consistent with the systematic review, as similar outcomes were identified. The systematic review demonstrates a high mortality rate (40%), alongside a poor AFS rate (44.1%).

Comparing the 30-day outcomes of COVID-19 ALI to surrounding literature on non-COVID-19 ALI emphasises the severity of the prognosis. A 10-year prospective study conducted by Howard et al. found a 30-day mortality of 24.7% in non-COVID-19 patients, which was significantly more favourable than the high mortality rate concluded in our case series of COVID-19 patients (57%) [18]. These results demonstrate that the 30-day outcomes in those with ALI are worse if the patient has COVID-19. Furthermore, the calculated geographical incidence of AT in the case series was 0.03%, whereas previous studies have shown that the incidence of ALI in the general non-COVID-19 population is 1:10,000. Even though the incidence is at a three-fold increase, it still remains low.

The case series ascertained that the location of AT differed in patients with COVID-19 as the majority of our sample developed thromboses involving more systemic, proximal, and thoracoabdominal vessels. This series reported that 21% of patients had a thrombus in their great vessels, 7% in the superior mesenteric artery, 36% in the proximal limbs, and 36% in the distal limbs. Cases affecting the major vessels including the aortic arch, descending thoracic aorta, mesenteric, splenic, and renal arteries have been associated with a poor prognosis [19,20]. Surrounding literature has shown frequent involvement of thrombosis in the upper limbs [21,22]. Conversely, AT has been predominately reported in the lower limb vessels of patients with COVID-19, particularly the distal aorta, iliac, superficial femoral, popliteal, and tibial (anterior and posterior) [19,20,23]. AT in the lower limbs has proven to be a greater thrombus burden with a poor prognosis.

When assessing the baseline characteristics of each patient, the series reported as high as 73% of patients were hospitalised due to severe infection. This indicates that severe COVID-19 is a significant risk factor for developing thromboembolic complications, which may be attributed to the aggressive nature of severe COVID-19, leading to the development of a cytokine storm and consequently, hypercoagulability [3]. Contrarily, a systematic review found the correlation between the incidence of ALI and the severity of COVID-19 to be debatable.

Veyre et al. reported the presence of ALI in a healthy 24-year-old with non-severe COVID-19 and no other predisposing conditions [24]. Similarly, 23.8% of ALI cases in Ilonzo et al.'s study had no respiratory symptoms with their positive COVID-19 result [11]. Incidentally, studies included in this systematic review often reported a background of atrial fibrillation in their patients, further supported by the case series that had a 40% background rate of atrial fibrillation. A study conducted by Hess et al. concluded that previous atrial fibrillation patients had a heightened risk of ALI, mirroring our results [25]. Furthermore, a single centre observational cohort study conducted by Bellosta et al. found a background of atrial fibrillation in all their participants [9].

Current guidelines recommend anticoagulation with heparin as the management of choice in patients with AT as it has shown to decrease mortality [6]. This study found a significantly high rate of death within 30 days. Of patients managed with anticoagulation, only 38% died. Of patients managed with palliative care, 100% died within 30 days as expected. Concerning the palliative group, the primary cause of death was COVID-19 in all but two patients. Conventional catheter embolectomy was used for patients who underwent revascularisation and was best performed under local anaesthetic. The results in the case series reflected a low 30-day AFS rate of 29% amongst the cohort, in line with a systematic review, which reports an AFS rate as low as 23.1% [17]. Despite four out of the seven reported studies having an AFS below 40%, the average AFS rate across all studies was slightly more favourable at 44.1%. Mascia et al. reported an AFS rate of 83.3%, which was significantly higher than any other study [13]. This may be due to the limited number of participants (n = 12) involved.

There are several limitations to this study. There was no standardised care protocol for COVID-19 patients referred to the vascular services. Consequently, a small number of our cohort was not evaluated using CTA imaging prior to management due to poor health; therefore, it is only possible to comment on the thrombus location in nine patients, lowering the internal validity of these results. It is likely that not every patient with AT was referred to the vascular services. During the first wave of the pandemic, in particular, staff were reluctant to operate on comorbid individuals, and due to the unprecedented stressors on the healthcare system, the management of severe COVID-19 was prioritised over the treatment of other comorbidities. As a result, some individuals may have been referred straight to ICU with severe COVID-19, rather than via vascular services and appropriately managed or palliated by the intensive care teams. In addition, this study was conducted during the first year of the pandemic prior to the availability and offering of the COVID-19 vaccination programme and the emergence of subsequent variants of the coronavirus. Hence, the study results may not be applicable to the current dominant variants, e.g., Omicron. The systematic review includes only small studies, with no large dataset for patients with COVID-19 and AT. There is a lack of data on patients' vaccination status and its impact on AT outcomes. There are also no randomised or non-randomised data regarding treatment options for AT.

## Conclusions

The hospital experience of COVID-19 and the review of surrounding literature gave similar results. The incidence of ALI still remains relatively low; however, the 30-day outcomes are poor. COVID-19 plays a vital role in viral-induced hypercoagulability and correlates with the severity of infection, as the majority of our patients were hospitalised. The data from our systematic review showed that COVID-19 was associated with a high incidence of amputation as well as a four-fold increase in mortality compared to non-COVID patients with ALI. Additionally, the majority of patients had more proximal locations of AT and therefore a more widespread CTA may be indicated.

## References

[1] Dormandy J, Heeck L, Vig S (1999). Acute limb ischemia. Semin Vasc Surg.

[2] (2022). Acute limb ischaemia. https://teachmesurgery.com/vascular/peripheral/acute-ischaemia/.

[3] Avila J, Long B, Holladay D, Gottlieb M (2021). Thrombotic complications of COVID-19. Am J Emerg Med.

[4] Lew TW, Kwek TK, Tai D (2003). Acute respiratory distress syndrome in critically ill patients with severe acute respiratory syndrome. JAMA.

[5] Bunce PE, High SM, Nadjafi M, Stanley K, Liles WC, Christian MD (2011). Pandemic H1N1 influenza infection and vascular thrombosis. Clin Infect Dis.

[6] (2022). National Institutes of Health. Coronavirus disease 2019 (COVID-19) treatment guidelines. https://www.covid19treatmentguidelines.nih.gov/.

[7] Moola S, Munn Z, Tufanaru C (2020). Chapter 7: systematic reviews of etiology and risk. JBI Manual for Evidence Synthesis.

[8] (2022). COVID-19 in the UK: how many coronavirus cases are there in my area?. https://www.bbc.co.uk/news/uk-51768274.

[9] Bellosta R, Luzzani L, Natalini G (2020). Acute limb ischemia in patients with COVID-19 pneumonia. J Vasc Surg.

[10] Goldman IA, Ye K, Scheinfeld MH (2020). Lower-extremity arterial thrombosis associated with COVID-19 is characterized by greater thrombus burden and increased rate of amputation and death. Radiology.

[11] Ilonzo N, Rao A, Berger K (2020). Acute thrombotic events as initial presentation of patients with COVID-19 infection. J Vasc Surg Cases Innov Tech.

[12] Indes JE, Koleilat I, Hatch AN (2021). Early experience with arterial thromboembolic complications in patients with COVID-19. J Vasc Surg.

[13] Mascia D, Kahlberg A, Melloni A, Rinaldi E, Melissano G, Chiesa R (2020). Single-center vascular hub experience after 7 weeks of COVID-19 pandemic in Lombardy (Italy). Ann Vasc Surg.

[14] Sánchez JB, Cuipal Alcalde JD, Ramos Isidro R (2021). Acute limb ischemia in a Peruvian cohort infected by COVID-19. Ann Vasc Surg.

[15] Tan CW, Fan BE, Teo WZ (2021). Low incidence of venous thrombosis but high incidence of arterial thrombotic complications among critically ill COVID-19 patients in Singapore. Thromb J.

[16] Al Raizah A, Al Askar A, Shaheen N (2021). High rate of bleeding and arterial thrombosis in COVID-19: Saudi multicenter study. Thromb J.

[17] Gonzalez-Fajardo JA, Ansuategui M, Romero C, Comanges A, Gómez-Arbeláez D, Ibarra G, Garcia-Gutierrez A (2021). Mortality of COVID-19 patients with vascular thrombotic complications. Med Clin (Engl Ed).

[18] Howard DP, Banerjee A, Fairhead JF, Hands L, Silver LE, Rothwell PM (2015). Population-based study of incidence, risk factors, outcome, and prognosis of ischemic peripheral arterial events: implications for prevention. Circulation.

[19] Etkin Y, Conway AM, Silpe J (2021). Acute arterial thromboembolism in patients with COVID-19 in the New York City area. Ann Vasc Surg.

[20] Kashi M, Jacquin A, Dakhil B, Zaimi R, Mahé E, Tella E, Bagan P (2020). Severe arterial thrombosis associated with COVID-19 infection. Thromb Res.

[21] Perini P, Nabulsi B, Massoni CB, Azzarone M, Freyrie A (2020). Acute limb ischaemia in two young, non-atherosclerotic patients with COVID-19. Lancet.

[22] Shao T, In-Bok Lee C, Jabori S, Rey J, Duran ER, Kang N (2020). Acute upper limb ischemia as the first manifestation in a patient with COVID-19. J Vasc Surg Cases Innov Tech.

[23] Anwar S, Acharya S, Shabih S, Khabut A (2020). Acute limb ischemia in COVID-19 disease: a mysterious coagulopathy. Cureus.

[24] Veyre F, Poulain-Veyre C, Esparcieux A, Monsarrat N, Aouifi A, Lapeze J, Chatelard P (2020). Femoral arterial thrombosis in a young adult after nonsevere COVID-19. Ann Vasc Surg.

[25] Hess CN, Huang Z, Patel MR (2019). Acute limb ischemia in peripheral artery disease: insights from EUCLID. Circulation.

